# Immobilized lipase catalyzing glucose stearate synthesis and their surfactant properties analysis

**DOI:** 10.1007/s13205-016-0501-z

**Published:** 2016-08-29

**Authors:** A. Maria Sebatini, Manisha Jain, P. Radha, S. Kiruthika, Krishnamurthi Tamilarasan

**Affiliations:** 1 Department of Biotechnology, School of Bioengineering, SRM University, Chennai, Tamilnadu 603203 India; 2 Department of Chemical Engineering, School of Bioengineering, SRM University, Chennai, Tamilnadu 603203 India

**Keywords:** Glucose stearate, Silica gel, Emulsification index, Antimicrobial activity

## Abstract

Sugar fatty acid esters are practical importance and have a variety of applications that include surfactants and as an emulsifying agent. In this study, we report glucose stearate synthesis using lipase-Fe_3_O_4_ nanoparticles catalyst. The influence of various reaction factors, such as silica gel concentration, molar ratio of sugar/acid, reaction temperature and speed of agitation on esterification by immobilized enzyme was analyzed. The glucose stearate esterification degree of 87.2 % was obtained under the optimized condition: 1:2 molar ratio of glucose/stearic acid, 2 % (w/v) of silica gel at 120 rpm and 40 °C. Glucose esters were characterized according to their surfactant activity like emulsification index, oil displacement activity and antimicrobial activity. The results indicated glucose stearate acts as biosurfactant, with emulsification index of 66 % in mustard oil and oil displacement activity of 19.64 cm^2^.

## Introduction

Biosurfactants are surface active substances, consisting both hydrophobic (lipids) and hydrophilic (sugar) portion on its molecules (Mulligan 2005). The main function of surfactants is to reduce surface and interface tensions between hydrophobic substances (oil, hydrocarbons and sterols) and hydrophilic water molecules (Desai and Banat 1997). In recent years, surfactants play an important role in medical, food, agricultural, cosmetic and bioremediation process industries (Amézcua et al. 2007; Dickinson 2009; Joshi et al. 2008; Nguyen et al. 2010; Rodrigues et al. 2006; Seydlová and Svobodová 2008).

Many carbohydrate esters synthesis by lipase enzyme, for instance, are used as antibacterial agents in food industry. The ester may be produced from renewable source and inexpensive substances under mild reaction conditions, which minimize side reactions compared to the chemical process (Nair et al. 2012). The environmental concern about chemical surfactants is promoting the exploration on the use of biosurfactant. In the past few years, there is a huge demand for biosurfactants over chemical surfactant because of its low toxicity to environment and biodegradability (Chamouleau et al. 2001; Nitschke and Csta 2007; Park et al. 2004; Tsavas et al. 2002). The application of chemically synthesized sugar esters is limited, because they are produced at high temperatures in toxic solvents and expensive purification is required. Even though the application of this surfactant is diversified, it is being limited due to problems associated with its preparation (Maja et al. 2008; Hill and Rhode 1999).

The nonionic biosurfactants of sugar ester are obtained from microbial and enzymatic process using renewable and inexpensive substrate (Atanu et al. 2008; Kshirsagar and Singhal 2007; Sabeder et al. 2006; Ye et al. 2010). Enzymatic process is quite favorable for sugar ester synthesis due to high specificity, high efficiency and lesser downstream process (Li et al. 2010; Plou et al. 2002; Sengupta et al. 2010). Many of the reports suggested that removal of water molecule during the esterification in reaction medium via molecular sieves, azeotropic distillation and pervaporation methods (Seydlová and Svobodová 2008). However, the industrial application of the biocatalysts is limited because of its high cost and difficult processing (Yong et al. 2008). To overcome the above limitations enzyme immobilization on supports materials was used (Ming et al. 2012; Yan et al. 2014; Hamidah et al. 2009). Immobilizations help for better dispersion of enzyme in reaction medium and also reduce enzyme contamination and facilitate easy separation of products (Badgujar et al. 2014).

In recent years, nanoparticles have been used as support material for lipase immobilization. Among the nanostructures, magnetic nanoparticles are low in toxicity and are easy to separate from reaction medium by applying a magnetic field (Jiang et al. 2009; Liu et al. 2011; Sohrabi et al. 2014). Recently, glucose esters synthesis by immobilized *Candida antarctica* lipase catalysis has been reported (Jiang et al. 2009). In the present work, we report lipase covalently immobilized on functionalized magnetic nanoparticles. We have investigated enzymatic synthesis of glucose ester using glucose and stearic acid as substrate. The effect of various reaction conditions for glucose ester synthesis was studied. We have also characterized the glucose esters by their surfactant and antimicrobial properties.

## Materials and methods

### Chemicals and microorganism

Microorganisms were obtained from MTCC, Institute of Microbial Technology (IMTECH), Chandigarh, India. All the media components used were of analytical grade, and were purchased from Hi Media Laboratories Pvt Ltd, (Mumbai, India). The solvents and stearic acid were purchased from Merck specialties Pvt Ltd, Mumbai, India. Lipase enzyme (*Rhizobium oryzae*) was purchased from sigma chemical, USA.

### Preparation of immobilized lipase enzyme

Magnetic nanoparticles (MNP) were prepared by co-precipitation method (Chia et al. 2012; Pan et al. 2009; Manisha et al. 2015). 400 mg of nanoparticles were dispersed in 0.4 g chitosan containing 20 mL of 1 % acetic acid solution. 1 N sodium hydroxide was slowly added to the reaction mixture to precipitate the chitosan coated MNP. 30 mg of lipase was added to 0.25 % *N*-(3-dimethylaminoproyl)-*N*-ethylcarbodiimide (EDC) containing 4 mL of 50 mM phosphate buffer solution (with 200 mM NaCl) and the solution was incubated at 25 °C for 1 h with shaking. Then, 30 mg of *N*-hydroxysuccinimide (NHS) was added to the solution and the incubation was continued for another 1 h. 50 mg of the chitosan coated MNP was added to this solution and incubated for further 4 h. The activated carboxyl groups of enzyme combined with amino group of nanoparticle by simple carbodiimide reaction to synthesized complex immobilized lipase (Manisha et al. 2016).

### Experimental setup

Glucose esters were synthesized by esterification reactions. The ester synthesis was carried out in glass vials by adding various molar ratio of glucose and stearic acid with silica gel in 25 mL of isooctane. The reaction mixture was stirred at 120 rpm for 10 min, later on reaction was initiated by adding 100 mg of immobilized lipase enzyme then kept at room temperature for 24 h. At the end of the reaction, the immobilized enzyme and silica gel were removed by filtration using filter paper with a pore size of 60 mm. Afterward, the sample was analysed to determine amount of ester content in the reaction mixture.

### Optimization of esterification condition of glucose ester with immobilized lipase

**Fig. 1:**
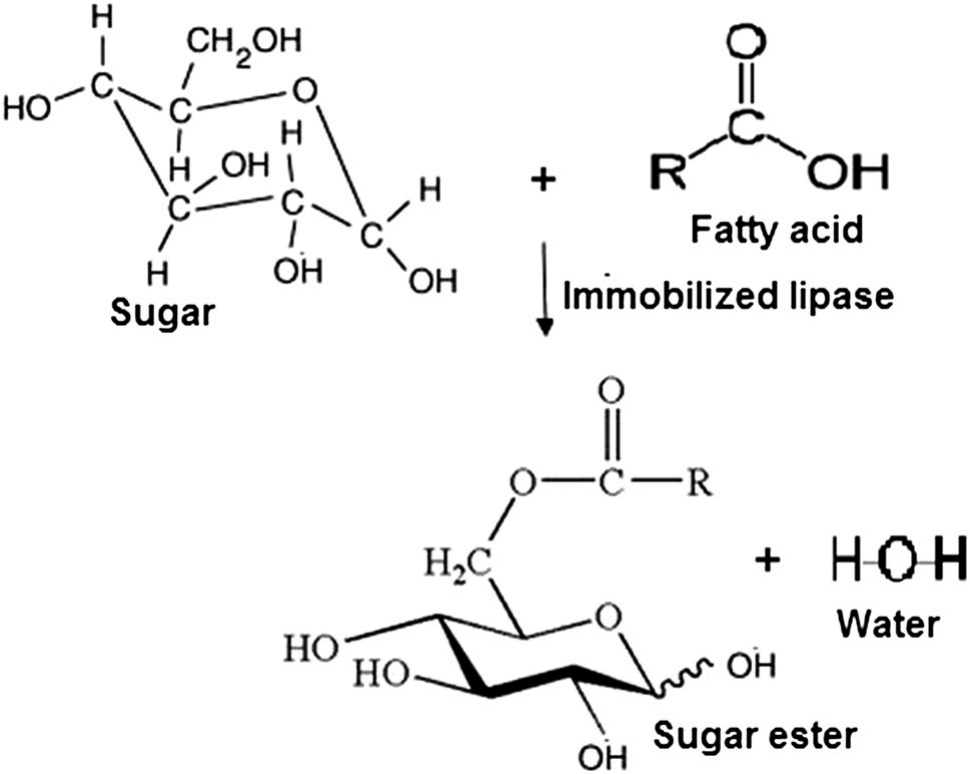
Lipase-catalyzed synthesis of glucose stearate ester

The experiments were conducted under 1:1–1:4 molar ratio of glucose to stearic acid, reaction temperature (20–60 °C) and speed of agitation (0–240 rpm) with 0–6 % (w/v) silica gel content. The sugar ester yield was analyzed using the sample withdrawn from the reaction mixtures. At the end of the esterification reaction, immobilized enzyme and unconverted substrate were separated by filtration and centrifugation methods. The filtrate containing the ester product was quantified by titration method. Figure 1 shows the scheme for lipase catalyzed synthesis of glucose stearate ester.

### Quantification of glucose ester

The glucose ester content was quantified by calculating the residual fatty acid amount in the reaction mixture, which was determined by the titration method (Leitgeb and Knez 1990). Briefly, 2 mL sample from the reaction mixture were titrated against 0.001 N standardized sodium hydroxide solution using phenolphthalein indicator. The yield was calculated using the formula:

1 $${\text{Yield }}\left( \% \right) \, = \, \left[ {100} \,-\,\right[{{X_{1} } \mathord{\left/ {\vphantom {{X_{1} } {X_{2} }}} \right. \kern-0pt} {X_{2} }}]] \, \times 100$$

$$X_{{1\left( {\text{sample}} \right)}} = \, V_{1} \times {N \mathord{\left/ {\vphantom {N {W_{1} }}} \right. \kern-0pt} {W_{1} }}$$

$$X_{{ 2 ( {\text{control)}}}} = \, V_{2} \times {N \mathord{\left/ {\vphantom {N {W_{2} }}} \right. \kern-0pt} {W_{2} }}$$

where *V*_1_ volume of sodium hydroxide used for sample, *V*_2_ volume of sodium hydroxide used for control, *N* normality of sodium hydroxide, *W*_1_ weight of sample, *W*_2_ weight of control. All the measurements were performed in triplicate and the results represent the standard deviation.

### Emulsification index and oil displacement activity of glucose ester

The emulsifying capacity of glucose stearate was analyzed by emulsification index (Leitgeb and Knez 1990; Nair et al. 2012). The emulsification index of ester sample was determined by adding oil and sugar ester (1:2 ratio) was homogenized using a vortex for 5 min. The emulsions were left to settle for 48 h, to measured height of the emulsion layer at 2 min, 24 and 48 h intervals. The emulsification index was calculated as the ratio of measured height of the emulsion layer to the total height of mixture and multiplying it by 100 (Nair et al. 2012). The oil displacement assay is a convenient method for surfactant activity studied (Morikawa et al. 1993). 30 mL of distilled water was added to a petri plate. 50 µL of oil was then added to the water surface followed by 10 µL of glucose ester (1 mg/mL) dropped on the center of oil surface. The occurrence of clear zone is an indication of the surfactant activity of ester. The activity of ester was directly proportional to oil displacement area in petri plate.

### Antimicrobial activity of glucose ester

The antimicrobial activity of glucose esters was tested against the *Bacillus subtilis*, *Bacillus cereus*, *Bacillus megaterium* and *E. coli* (Manuel et al. 2005; Vagi et al. 2005). The activity of esters was tested in nutrient liquid media for the growth of microorganism. The stock solution of sugar ester (5 mg/mL) was prepared to add in the liquid media. 100 µL of overnight culture microorganism was used as inoculate for test medium and incubation for 24 h at 37 °C. Antimicrobial activity was tested by measuring the turbidity of growth using UV–vis spectrophotometer at 600 nm. The growth of micro-organisms in the medium containing defined concentration of sugar fatty acid ester were compared with those obtained in a medium without sugar fatty acid ester (control) (Maja et al. 2008). The percentage of inhibition of microorganism in test medium was calculated compared to growth of control medium.

## Results and discussion

### Effect of silica gel content

**Fig. 2:**
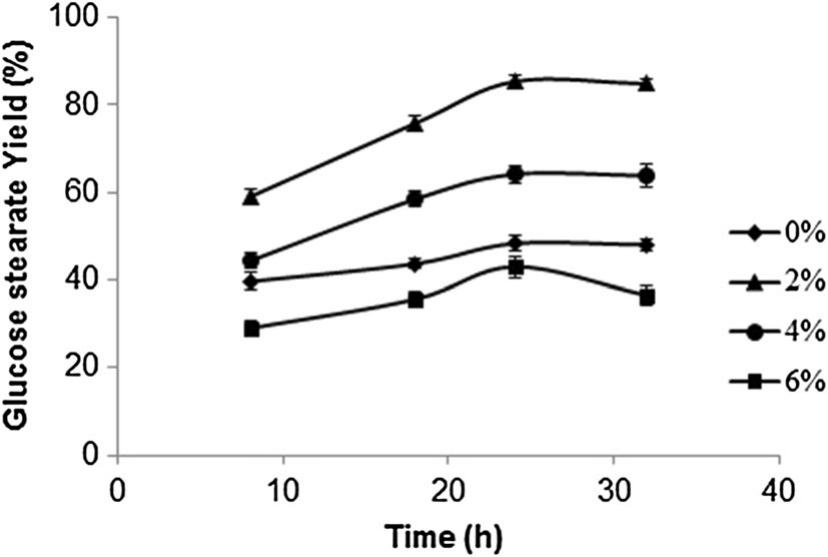
Effect of silica gel concentration on glucose stearate synthesis

In esterification reaction, water content in the reaction mixture affects the lipase activity and favors the equilibrium state (Chortyk 1996). Therefore, the effect of water content in reaction medium for enzymatic synthesis of glucose ester was measured. In this work esterification reaction were carried out with various concentrations (0–6 % w/v) of silica gel adsorbent was studied. Silica gel not only dried the reaction medium but also shifted the equilibrium to the synthesis of glucose ester by adsorbing the water molecule (Jiugao et al. 2008). As shown in Fig. 2, the concentration of silica gel increased to 2 %, the esterification increased up to 75 %, but on further increase in silica gel concentration, the esterification decreased significantly. It might be due to adsorption of the minimal water necessary for the enzyme activity in the reaction. From the result maximum conversion (85.4 %) of glucose stearate was obtained, when 2 % silica gel was used.

### Effect of temperature and speed of agitation

**Fig. 3:**
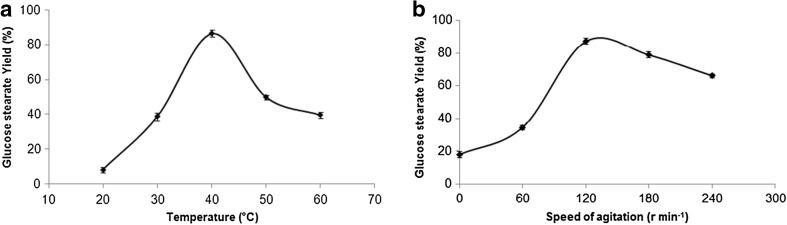
**a** Effect of temperature on glucose stearate synthesis, **b** effect of speed of agitation on glucose stearate synthesis

Temperature is an important parameter for any enzymatic reaction that increases the molecular collision and improves the substrate solubility in reaction media (Badgujar et al. 2013). The effect of reaction temperature (20–60 °C) on esterification was tested. As shown in Fig. 3a, the lower reaction temperature resulted in poor conversion because of the increase in viscosity of medium that led to higher mass transfer resistance. When reaction temperature reached higher than 40 °C, the esterification decreased slowly, because of the equilibrium of the reaction and the loss of enzyme activity. From the results the highest esterification 86 % was obtained at 40 °C.

The effect of external mass transfer limit was analyzed by varying the speed of agitation, which is imperative for the enzymatic reaction. This is a micro aqueous solid–liquid system in which reactants are in liquid phase while enzyme is in solid phase (Badgujar et al. 2013). The effect of speed of agitation on esterification reaction was examined in solvent medium (Fig. 3b). The conversion of glucose stearate increased with increasing speed of agitation from 60–120 rpm and then decreased slowly. High esterification (88 %) was obtained at 120 rpm after 24 h incubation. These results shows that mass transfer effect was observed in between the 60 to 120 rpm; as soon as mass transfer barrier is reached then there is no influence of the mass transfer diffusion on the reaction rate and after 220 rpm there was no significant increase in the activity (Yadav and Pawar 2012).

### Effect of substrate molar ratio

**Fig. 4:**
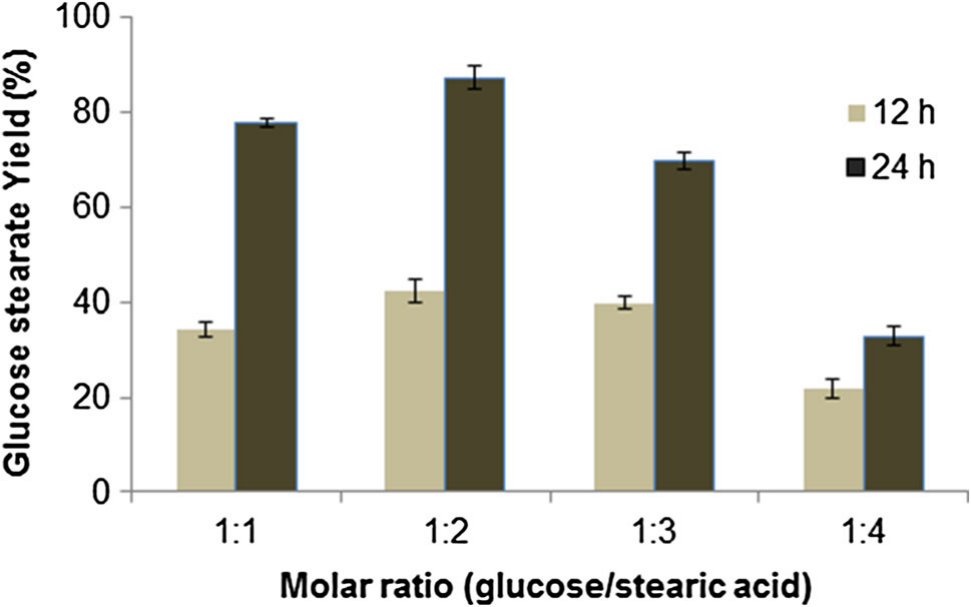
Effect of molar ratio of sugar/fatty acid on glucose stearate synthesis

The effect of stearic acid concentration on glucose stearate synthesis was examined at constant glucose concentration. In a set of experiments, glucose was kept constant at 1 mM and the quantity of stearic acid was varied as 1:1, 1:2, 1:3 and 1:4 mM. The esterification rate increased up to 1:2 ratio, after that the esterification rate decreased (Fig. 4). The highest conversion of 87 % was obtained at the glucose/stearic acid molar ratios of 1:2. Lower the molar ratio of stearic acid, probability to get low active sites then difficult to catalyze reaction. At higher molar ratio of stearic acid, lower solubility in organic solvent destroys the balance of esterification, and the reaction is hindered. Similar type of glucose ester was synthesized by an immobilized lipase with 0.8 g molecular sieves/2 mL acetone at 40 °C for 48 h, and the 38 % acid conversion was obtained (Jiugao et al. 2008). In addition, a higher stearic acid concentration may change the catalytic environment and the active site of immobilized enzyme (He et al. 2010).

### Reusability of the immobilized lipase

**Fig. 5:**
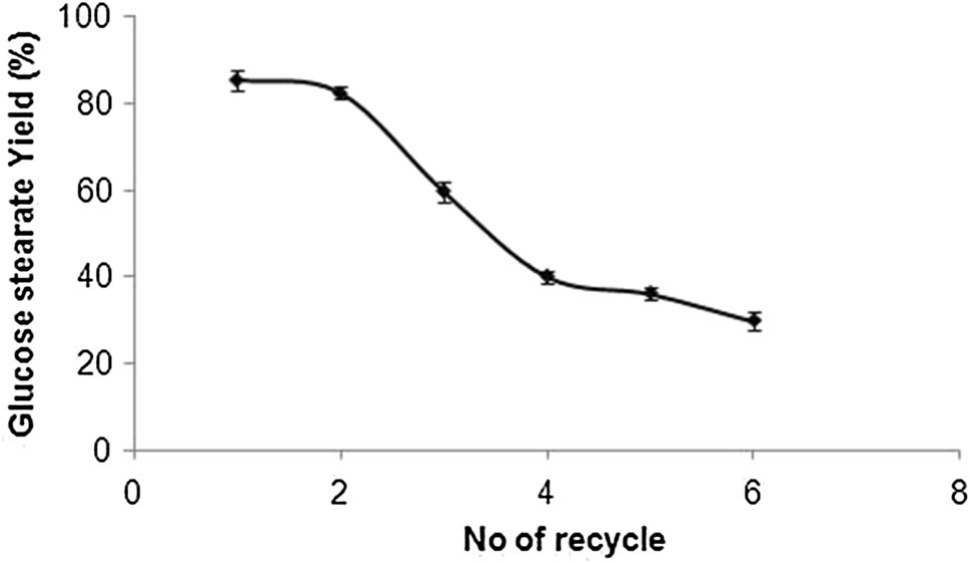
Reusability of the immobilized lipase for glucose stearate synthesis

Reusability of the immobilized enzyme is relatively important for its industrial application. To investigate the reusability, immobilized enzyme was first washed with ethanol and then with deionized water after one reaction cycle and reintroduced into a new esterification reaction. The effect of repeated use of immobilized enzyme on esterification of glucose is shown in Fig. 5. The results were observed that the esterification was still retained (60 %) after the three reuses. After using three times, the conversion marginally decreased and it was due to the denaturation of the enzyme on MNP support during separation from reaction mixture.

### Glucose stearate ester production

**Fig. 6:**
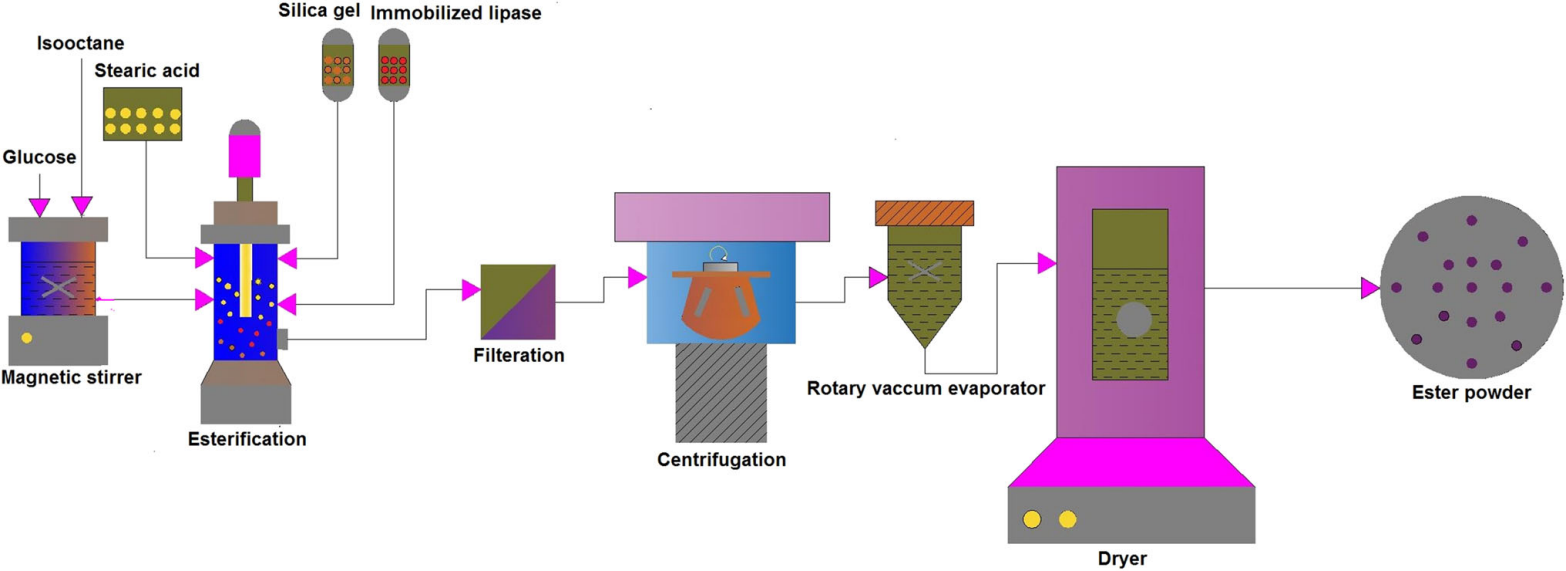
Schematic diagrams of glucose stearate synthesis and purification process

The reaction mixture consists of 2 mM glucose and 4 mM stearic acid (1:2 molar ratio of sugar and acid) dissolved in 50 mL of isooctane with 500 mg of immobilized lipase. To improve conversion, 100 mg silica gel was added to adsorb water generated from the reaction mixture during esterification. The reaction mixture was incubated at 40 °C, 120 rpm for 48 h. At the end of the esterification reaction, reaction mixture was separated by filtration and centrifugation methods. The filtrate contains ester product; it was concentrated by rotary vacuum evaporator. A schematic diagram of the overall process of glucose ester synthesis in batch process is shown in Fig. 6.

### Glucose stearate structural analysis

**Fig. 7:**
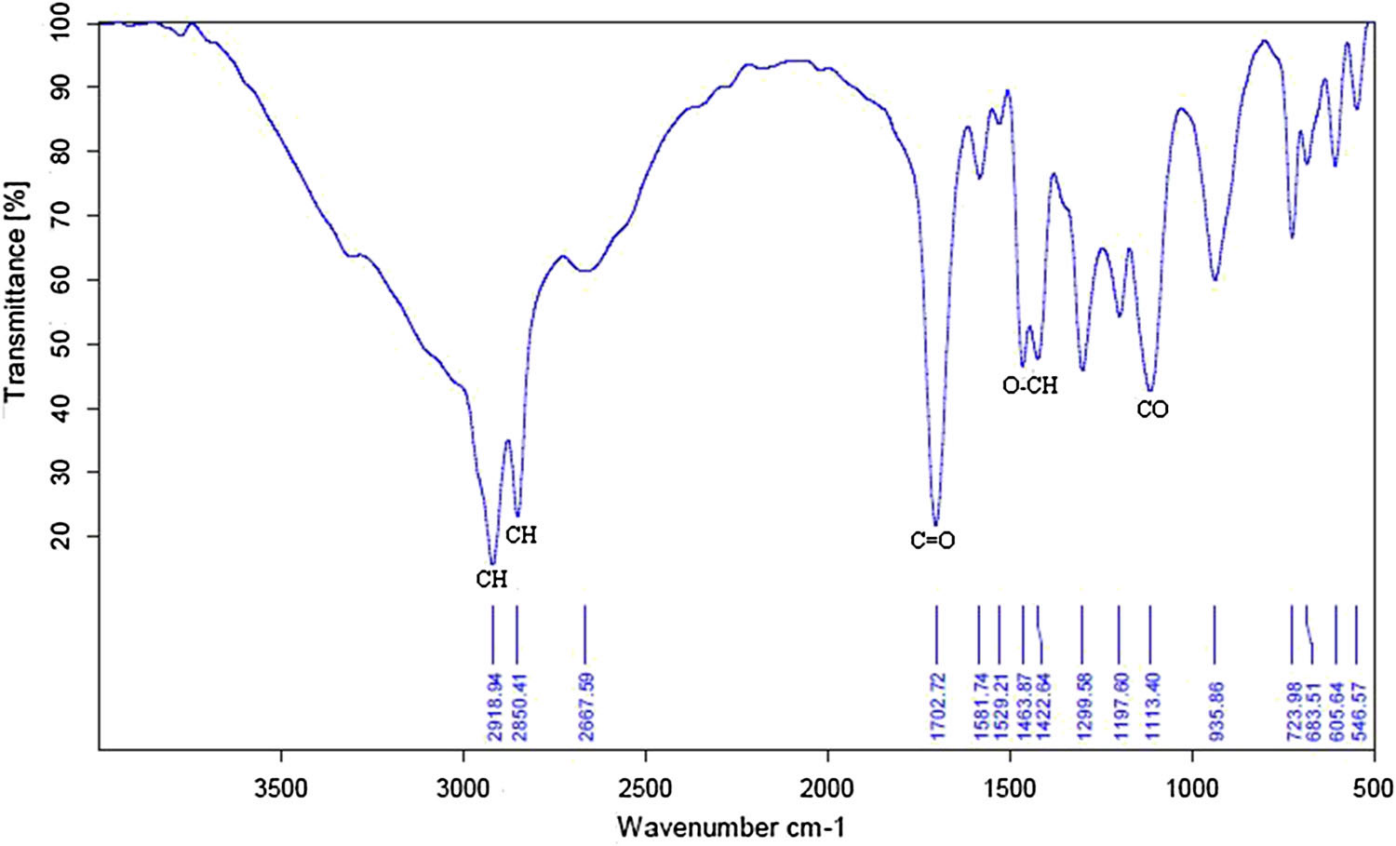
Fourier-transform infrared spectra of glucose stearate

The Fourier transform-IR spectrum chart of purified glucose stearate is shown in Fig. 7. The observed band characteristic of C–O (1113 cm^−1^) indicated that glucose stearate contained some sugars. The C=O stretching is calculated from 1702 cm^−1^ and combination band of OCH and COH (1463, 1422 cm^−1^) and C–H (2918, 2850 cm^−1^) can be observed. Similar results were obtained by other authors (Jiugao et al. 2008) when determining the chemical structure of 6-*O*-glucose stearate synthesized by *Candida* lipase.

### Oil displacement assay

**Fig. 8:**
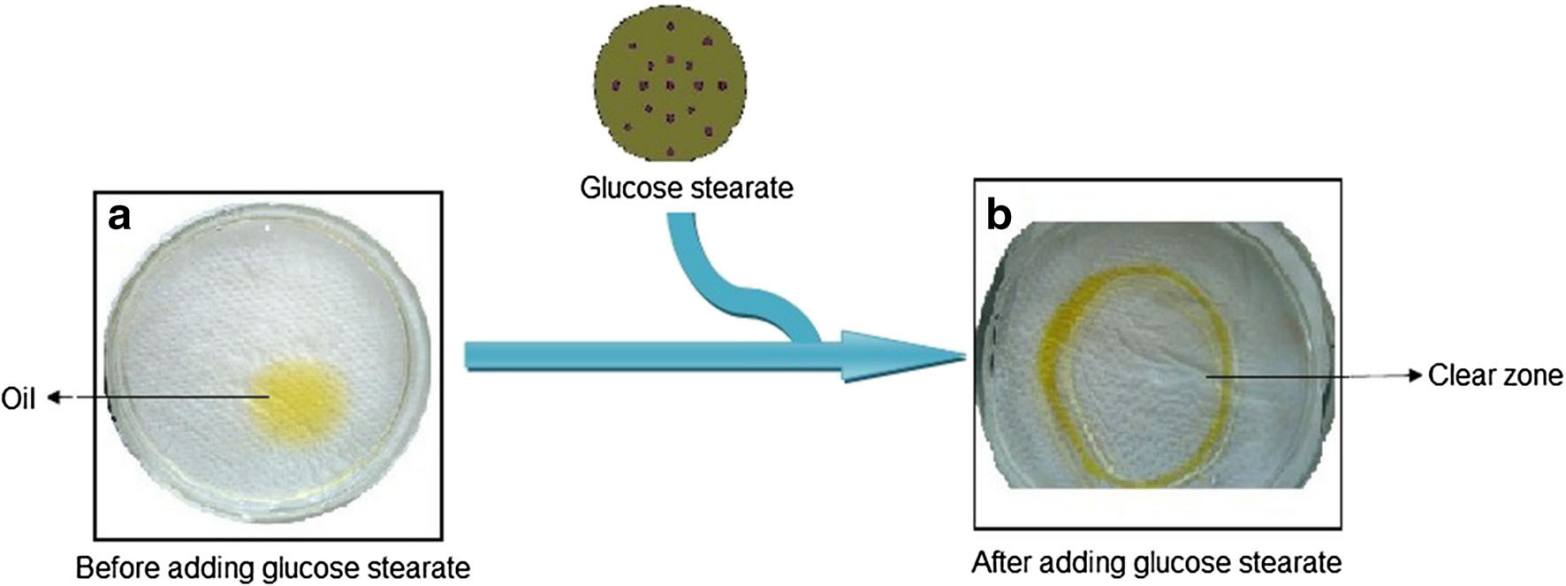
Oil displacement activity of glucose stearate. **a** Before adding glucose stearate and **b** after adding glucose stearate

Oil displacement assay is a more sensitive analysis for surface active compounds (Mulligan 2005). Oil displacement activity of ester attributed to the formation of critical micelle concentration which helps to reduce the surface tension between two interfaces. In this study oil displacement activity was measured by the area of clear zone formed on the oil–water surface (Fig. 8). 19.64 m^2^ clear zone was formed after adding 1 mg/mL of glucose ester on the surface of oil drop. The reduction in the surface tension was observed to formation of clear zone at the surface. The zone area formation is directly proportional to surfactant concentration.

### Emulsification index

**Fig. 9:**
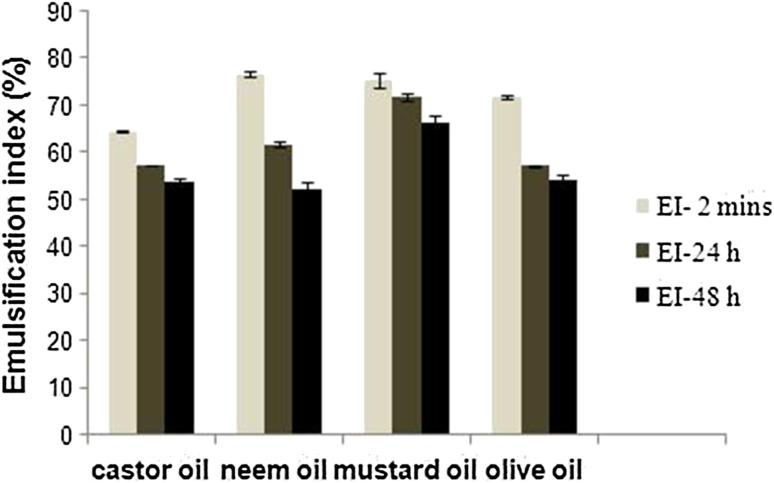
Emulsification index of glucose stearate

Emulsion index was studied at 2 min, 24 and 48 h time intervals with 1:2 (glucose ester: oil sample) ratio mixture. The comparison emulsification results of different oil are shown in Fig. 9. The highest emulsification indexes of 71 and 61 % were obtained from mustard oil and neem oil, respectively, at 24 h. Emulsification indexes of olive oil and castor oil was calculated to be 57.2 and 57.1 %, respectively. Higher emulsification index represents higher stability. Sugar ester achieved highest stability (EI 66 %) in mustard oil at 48 h (Nitschke and Csta 2007).

### Antimicrobial activity of sugar ester

**Fig. 10:**
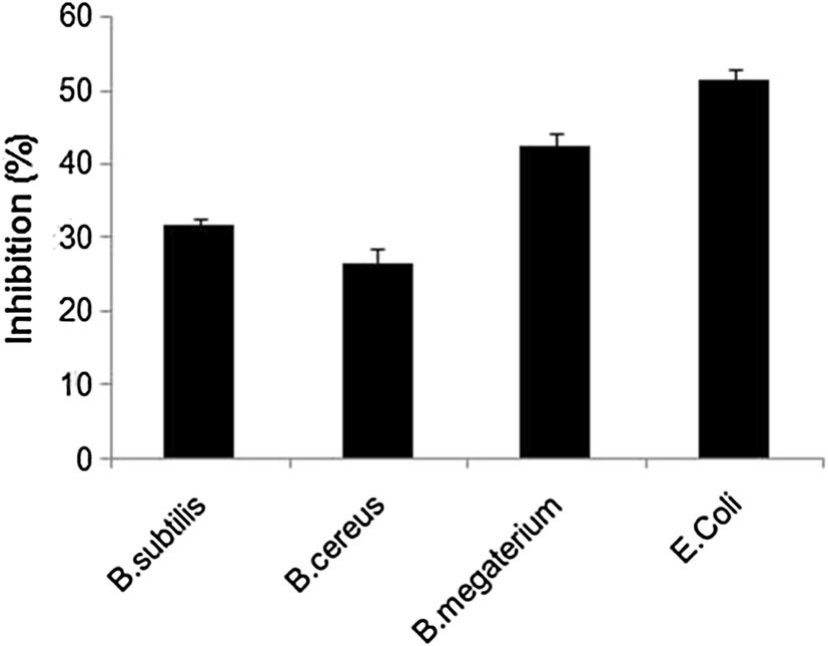
Growth inhibition activity of glucose stearate

Sugar esters are valuable compound in food industry as antibacterial agents because it is biodegradable and nontoxic. Most of the previous studies on antimicrobial properties of commercial sugar esters were tested against different microorganisms (Plou et al. 2002; Hathcox and Beuchat 1996; Tsuchido et al. 1993). Our enzymatically synthesized glucose stearate antimicrobial properties were studied against various bacterial species as shown in Fig. 10. Among the bacteria tested, *Bacillus subtilis*, *Bacillus cereus* and *Bacillus megaterium* was inhibited 31, 26 and 42 %, respectively, compare to original growth. In addition, glucose ester showed strong inhibition (52 %) of *E. coli* growth at 1 mg/mL concentration. Similarly, other reported that sucrose monolaurate strongly inhibits the growth of *E. coli* in concentration of 1 mg/ml (Kato and Arima 1971). But pervious report show that about 10 % higher inhibition against *Bacillus cereus* was obtained after 24 h of growth, when enzymatically synthesized sugar ester.

## Conclusion

We prepared lipase immobilized nanoparticles and employed it as a biocatalyst for sugar ester synthesis. The optimal reaction conditions that achieved highest esterification rate (87.2 %) were as follows: 1:2 molar ratio (glucose: stearic acid) with 2 % (w/v) silica gel at 40 °C, and 120 rpm. Furthermore, the glucose ester showed better surfactant properties with 67 % of emulsification index (EI) and 19.64 cm^2^ oil displacement activities. Glucose ester showed highest antibacterial activity against *E. coli* with 50 % of inhibition.
